# Spatial distribution of lipid droplets during starvation: Implications for lipophagy

**DOI:** 10.1080/19420889.2016.1183854

**Published:** 2016-06-24

**Authors:** Antonio Daniel Barbosa, Symeon Siniossoglou

**Affiliations:** Cambridge Institute for Medical Research, University of Cambridge, Cambridge, UK

**Keywords:** lipid droplets, lipophagy, nuclear membrane, nucleus-vacuole junction

## Abstract

Survival during starvation depends largely on metabolic energy, which is stored in the form of neutral lipids in specialized organelles known as lipid droplets. The precursors for the synthesis of neutral lipids are also used for membrane biogenesis, which is required for cell growth and proliferation. Therefore cells must possess mechanisms to preferentially channel lipid precursors toward either membrane synthesis or lipid droplet storage, in response to nutrient status. How this partitioning is spatially regulated within the endoplasmic reticulum (ER) where lipid droplets co-localize, remains poorly understood. We have recently shown that at the onset of starvation lipid droplets concentrate at a perinuclear ER subdomain flanking the nucleus-vacuole junction (NVJ) and that this is crucial for maintaining proper nuclear shape and ER membrane organization. Here we show that disruption of the NVJ does not block the translocation and internalization of lipid droplets into the vacuole for their degradation, which takes place at later stages of starvation. We propose that alternative pathways of lipid droplet translocation from the ER to the vacuole may exist to enable stationary phase-induced lipophagy.

Eukaryotic cells store fatty acids (FAs) as inert triacylglycerols or steryl esters (often referred to as neutral lipids) in ubiquitous organelles called lipid droplets (LDs).^1^ It is generally accepted that LDs emerge from the endoplasmic reticulum (ER) and in most cell types remain closely associated with it.^1^ Neutral lipid storage serves multiple functions in cells: (a) it prevents lipotoxicity of free FAs,^2^ (b) provides a source of energy during starvation,^3^ and (c) supplies lipid precursors for phospholipid synthesis that can fuel membrane biogenesis during growth and proliferation.^4^ Neutral lipid-derived precursors have been also proposed to play a role in autophagy during starvation, by controlling autophagosome membrane formation^5,6^ or ER membrane homeostasis.^7^ While the storage of FAs into neutral lipids is intrinsically associated with LD formation, the role of LDs in energy production and membrane synthesis relies on the regulated release of its stored FAs and other lipid intermediates in response to nutritional or environmental signals. This mobilization can result from either lipolysis or lipophagy.

In *Saccharomyces cerevisiae*, lipolysis is mediated by the LD-associated triacylglycerol lipases Tgl3, Tgl4 and Tgl5 and the steryl ester lipases Tgl1, Yeh1 and Yeh2.^3^ In contrast, lipophagy resembles microautophagy and requires core components of the autophagic machinery.^8,9^ To date, 2 conditions are known to induce lipophagy: nitrogen starvation and stationary phase (stat-phase lipophagy).^8,9^ Interestingly, distinct lipid microdomains are formed on the vacuolar membrane during stationary phase: a liquid-disorded domain and a sterol-enriched liquid-ordered domain.^10^ During stat-phase lipophagy, the internalization of LDs by the vacuole depends on their association with the sterol microdomain.^9^ Notably, nitrogen starvation does not induce the formation of microdomains,^10^ suggesting that the interaction of LDs with the vacuolar membrane is not the same in the 2 types of lipophagy.

The subsequent release of lipid constituents from internalized LDs during nitrogen starvation-induced lipophagy requires the putative lipase Atg15, which also degrades other autophagic and cytoplasm to vacuole targeting (Cvt) bodies. This activity is important for growth under conditions of compromised *de novo* biosynthesis of FAs.^8^ It is likely that Atg15 plays a similar role during Stat-phase lipophagy. In fact, lipophagy itself is required for the formation of the microdomains – probably due to the release of sterols from steryl-esters – and the domains are absent in the *atg15*Δ mutant.^9^ Remarkably, lipolysis mediated by Tgl3 and Tgl4 increases in cells lacking Atg15,^11^ suggesting a cross talk between lipophagy and lipolysis to regulate neutral lipid levels.

Since LDs must interact with the vacuolar membrane to be internalized, their spatial organization at the onset of lipophagy may define the mechanism of neutral lipid mobilization. We have recently shown that, as cells face starvation during glucose exhaustion, the biogenesis of LDs takes place at the perinuclear ER flanking the nuclear vacuole junctions (NVJ),^12^ a membrane contact site established by the physical interaction of Nvj1 (on the perinuclear-ER) and Vac8 (on the vacuolar membrane).^13^ LD biogenesis at this specific subdomain of the perinuclear ER is a consequence of the recruitment at this site of Pah1, a Mg^2^^+^-dependent phosphatidate phosphatase that plays a major role in triglyceride synthesis.^14^ Interestingly, glucose depletion triggers intracellular acidification^15^ that, in turn, controls important events in neutral lipid metabolism: (1) it increases the activity of Nem1-Spo7,^16^ a transmembrane phosphatase complex that activates Pah1;^17,18^ and (2) stimulates the formation of the vacuolar microdomains required for stat-phase lipophagy.^10^ Moreover, Pah1 localization to NVJ is concomitant with the early-steps of vacuolar membrane microdomain formation, which is prevented by the deletion of *NEM1*.^10^ Together, these results suggest that phosphatidic acid or its conversion into diacylglycerol by Pah1 may be involved in the formation of the microdomains.

During stat-phase lipophagy, LDs must migrate from the perinuclear ER to the vacuolar membrane. It is, therefore, reasonable to speculate that, by keeping the nucleus and vacuole in close proximity, NVJ may promote the movement of LDs. In fact, *NVJ1* mRNA levels increase and the contact site enlarges in stationary phase.^19^ To test this hypothesis, we analyzed lipophagy in cells expressing Vph1-mCherry - a vacuolar protein that partitions in the liquid-disordered microdomains^10^ - stained with the neutral lipid dye BODIPY 493/503 during stationary phase in cells lacking Nvj1. As previously reported,^10^ deletion of *NVJ1* does not compromise the formation of the vacuolar membrane microdomains. For example, *nvj1*Δ cells show the distinctive quasi-symmetrical segregation of Vph1-mCherry (Fig. 1, see inset for *nvj1*Δ cells). As in wild-type cells, in cells lacking *NVJ1* a clear accumulation of BODIPY-stained LDs is observed in the vacuole (Fig. 1, day 4). Similar results were also obtained in cells deleted for *ATG15* that, by preventing the degradation of LDs in the vacuole, facilitate the detection of lipid (Fig. 1, day 4).

**Figure 1.**
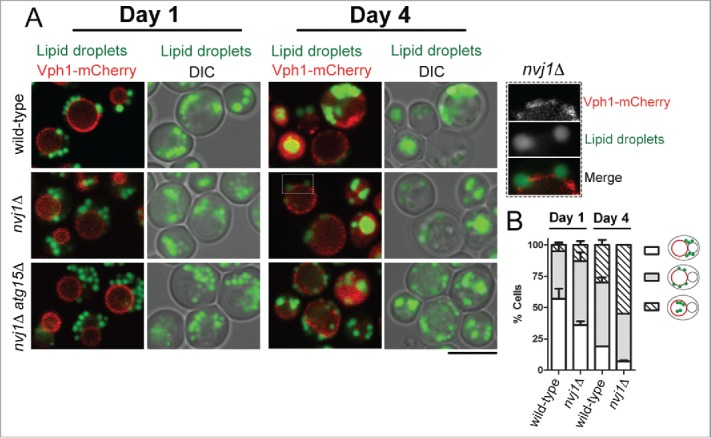
Deletion of *NVJ1* does not disrupt lipophagy in budding yeast. (A) Wild-type (RS453), and the isogenic *nvj1*Δ (*nvj1*::*HIS3MX6*) and *nvj1*Δ *atg15*Δ (*nvj1*::*HIS3MX6 atg15*::*TRP1*) strains expressing a chromosomally integrated *VPH1-mCherry* fusion were grown in synthetic medium as previously described.^12^ Cultures were inoculated at OD_600_ of 0.1 and imaged after 1 or 4 d of continuous growth at 30°C. LDs were labeled with BODIPY 493/503 as previously described.^12^ Cells were imaged using a Zeiss LSM880 confocal microscope and the ZEN2 software. Cells were visualized from the periphery by taking 10 optical sections, each 0.8 μm thick. A single mid-section is shown in all panels. At day 1, LDs concentrate at one side of the vacuole that is in contact with the nucleus. ^12^ At day 4, LDs associate with vacuolar membrane domains that are devoid of Vph1-mCherry (see magnified inset for *nvj1*Δ cells). At this stage, and similar to the wild-type, many *nvj1*Δ and *nvj1*Δ *atg15*Δ cells display strong vacuolar BODIPY signal indicating the presence of internalized LDs. Bar, 5 μm. (B) Quantification of LD distribution shown in wild-type and *nvj1*Δ cells shown in A from 3 independent experiments. The schematic on the right depicts the LD distribution patterns quantified. Red, vacuole; green, LDs.

Previous work has shown that loss of Nvj1 disrupts the nucleus-vacuole contact.^13^ The presence of stat-phase lipophagy in *nvj1*Δ cells, however, does not exclude a role for other ER-vacuole contact sites in the passage of LDs to the vacuole. Recent studies have identified additional protein components of ER-vacuole contact sites, which are not restricted to the perinuclear ER;^20-22^ moreover ER-vacuole contacts are still present in cells lacking *NVJ1*.^20,22^ Given that in yeast LDs remain closely associated with the ER throughout their life cycle,^23-25^ parallel pathways for their translocation from the ER to the vacuole during starvation are likely to exist. This may explain the increase in lipophagy observed in *nvj1*Δ cells (Fig. 1B), which could result from a compensatory mechanism of the other ER-vacuole contact sites due to the loss of NVJ.

The spatial organization of FA storage may have also important implications for the regulation of organelle structure. Recruitment of Pah1 in close proximity to LDs ensures that its product, diacylglycerol, is acylated with FAs so that the resulting triacylglycerol is efficiently stored in LDs. Such metabolic channeling is important because diacylglycerol can be also used for phospholipid synthesis. Consistent with this model, in the absence of LDs or triacylglycerol-synthesizing enzymes, Pah1 is still recruited to the nuclear membrane but diacylglycerol is redirected toward phospholipids, resulting in nuclear shape deformation and ER membrane expansion.^12^ We speculate that the presence of LDs within the various ER membrane domains and their association with other organelles may be crucial not only for energy storage and mobilization but also for controlling lipid flux to membranes and consequently, organelle dynamics.

## References

[1] Pol A, Gross SP, Parton RG. Biogenesis of the multifunctional lipid droplet: lipids, proteins, and sites. J Cell Biol 2014; 204:635-46; PMID:24590170; http://dx.doi.org/10.1083/jcb.201311051 24590170 PMC3941045

[2] Garbarino J, Sturley SL. Saturated with fat: new perspectives on lipotoxicity. Curr Opin Clin Nutr Metab Care. 2009; 12:110-6; PMID:19202381; http://dx.doi.org/10.1097/MCO.0b013e32832182ee 19202381

[3] Athenstaedt K, Daum G. The life cycle of neutral lipids: synthesis, storage and degradation. Cell Mol Life Sci 2006; 63:1355-69; PMID:16649142; http://dx.doi.org/10.1007/s00018-006-6016-8 16649142 PMC11136409

[4] Kurat CF, Wolinski H, Petschnigg J, Kaluarachchi S, Andrews B, Natter K, Kohlwein SD. Cdk1/Cdc28-dependent activation of the major triacylglycerol lipase Tgl4 in yeast links lipolysis to cell-cycle progression. Mol Cell 2009; 33:53-63; PMID:19150427; http://dx.doi.org/10.1016/j.molcel.2008.12.019 19150427

[5] Dupont N, Chauhan S, Arko-Mensah J, Castillo EF, Masedunskas A, Weigert R, Robenek H, Proikas-Cezanne T, Deretic V. Neutral lipid stores and lipase PNPLA5 contribute to autophagosome biogenesis. Curr Biol 2014; 24:609-20; PMID:24613307; http://dx.doi.org/10.1016/j.cub.2014.02.008 24613307 PMC4016984

[6] Shpilka T, Welter E, Borovsky N, Amar N, Mari M, Reggiori F, Elazar Z. Lipid droplets and their component triglycerides and steryl esters regulate autophagosome biogenesis. EMBO J 2015; 34:2117-31; PMID:26162625; http://dx.doi.org/10.15252/embj.201490315 26162625 PMC4557665

[7] Velázquez AP, Tatsuta T, Ghillebert R, Drescher I, Graef M Lipid droplet-mediated ER homeostasis regulates autophagy and cell survival during starvation. J Cell Biol 2016; 212:621-631; http://dx.doi.org/10.1083/jcb.201508102 26953354 PMC4792078

[8] van Zutphen T, Todde V, De Boer R, Kreim M, Hofbauer HF, Wolinski H, Veenhuis M, van der Klei IJ, Kohlwein SD. Lipid droplet autophagy in the yeast *Saccharomyces cerevisiae*. Mol Biol Cell 2014; 25:290-301; PMID:24258026; http://dx.doi.org/10.1091/mbc.E13-08-0448 24258026 PMC3890349

[9] Wang CW, Miao YH, Chang YS. A sterol-enriched vacuolar microdomain mediates stationary phase lipophagy in budding yeast. J Cell Biol 2014; 206:357-66; PMID:25070953; http://dx.doi.org/10.1083/jcb.201404115 25070953 PMC4121974

[10] Toulmay A, Prinz WA. Direct imaging reveals stable, micrometer-scale lipid domains that segregate proteins in live cells. J Cell Biol 2013; 202:35-44; PMID:23836928; http://dx.doi.org/10.1083/jcb.201301039 23836928 PMC3704982

[11] Maeda Y, Oku M, Sakai Y. A defect of the vacuolar putative lipase Atg15 accelerates degradation of lipid droplets through lipolysis. Autophagy 2015; 11:1247-58; PMID:26061644; http://dx.doi.org/10.1080/15548627.2015.1056969 26061644 PMC4590595

[12] Barbosa AD, Sembongi H, Su WM, Abreu S, Reggiori F, Carman GM, Siniossoglou S. Lipid partitioning at the nuclear envelope controls membrane biogenesis. Mol Biol Cell 2015; 26:3641-57; PMID:26269581; http://dx.doi.org/10.1091/mbc.E15-03-0173 26269581 PMC4603934

[13] Pan X, Roberts P, Chen Y, Kvam E, Shulga N, Huang K, Lemmon S, Goldfarb DS. Nucleus-vacuole junctions in Saccharomyces cerevisiae are formed through the direct interaction of Vac8p with Nvj1p. Mol Biol Cell 2000; 11:2445-57; PMID:10888680; http://dx.doi.org/10.1091/mbc.11.7.2445 10888680 PMC14931

[14] Han GS, Wu WI, Carman GM. The *Saccharomyces cerevisiae* Lipin homolog is a Mg^2+^-dependent phosphatidate phosphatase enzyme. J Biol Chem 2006; 281:9210-8; PMID:16467296; http://dx.doi.org/10.1074/jbc.M600425200 16467296 PMC1424669

[15] Martínez-Muñoz GA, Kane P Vacuolar and plasma membrane proton pumps collaborate to achieve cytosolic pH homeostasis in yeast. J Biol Chem 2008; 283:20309-19; http://dx.doi.org/10.1074/jbc.M710470200 18502746 PMC2459297

[16] Su WM, Han GS, Carman GM. Yeast Nem1-Spo7 protein phosphatase activity on Pah1 phosphatidate phosphatase is specific for the Pho85-Pho80 protein kinase phosphorylation sites. J Biol Chem 2014; 289:34699-708; PMID:25359770; http://dx.doi.org/10.1074/jbc.M114.614883 25359770 PMC4263874

[17] Santos-Rosa H, Leung J, Grimsey N, Peak-Chew S, Siniossoglou S. The yeast lipin Smp2 couples phospholipid biosynthesis to nuclear membrane growth. EMBO J 2005; 24:1931-41; PMID:15889145; http://dx.doi.org/10.1038/sj.emboj.7600672 15889145 PMC1142606

[18] Karanasios E, Han GS, Xu Z, Carman GM, Siniossoglou S. A phosphorylation-regulated amphipathic helix controls the membrane translocation and function of the yeast phosphatidate phosphatase. Proc Natl Acad Sci USA 2010; 107:17539-44; PMID:20876142; http://dx.doi.org/10.1073/pnas.1007974107 20876142 PMC2955120

[19] Roberts P, Moshitch-Moshkovitz S, Kvam E, O'Toole E, Winey M, Goldfarb DS. Piecemeal microautophagy of nucleus in *Saccharomyces cerevisiae*. Mol Biol Cell 2003; 14:129-41; PMID:12529432; http://dx.doi.org/10.1091/mbc.E02-08-0483 12529432 PMC140233

[20] Murley A, Sarsam RD, Toulmay A, Yamada J, Prinz WA, Nunnari J. Ltc1 is an ER-localized sterol transporter and a component of ER-mitochondria and ER-vacuole contacts. J Cell Biol 2015; 209:539-48; PMID:25987606; http://dx.doi.org/10.1083/jcb.201502033 25987606 PMC4442815

[21] Gatta AT, Wong LH, Sere YY, Calderón-Noreña DM, Cockcroft S, Menon AK, Levine TP. A new family of StART domain proteins at membrane contact sites has a role in ER-PM sterol transport. Elife 2015; 4: e07253; PMID:26001273; http://dx.doi.org/10.7554/eLife.07253 26001273 PMC4463742

[22] Henne WM, Zhu L, Balogi Z, Stefan C, Pleiss JA, Emr SD. Mdm1/Snx13 is a novel ER-endolysosomal interorganelle tethering protein. J Cell Biol 2015; 210:541-51; PMID:26283797; http://dx.doi.org/10.1083/jcb.201503088 26283797 PMC4539980

[23] Jacquier N, Choudhary V, Mari M, Toulmay A, Reggiori F, Schneiter R. Lipid droplets are functionally connected to the endoplasmic reticulum in Saccharomyces cerevisiae. J Cell Sci 2011; 124:2424-37; PMID:21693588; http://dx.doi.org/10.1242/jcs.076836 21693588

[24] Wolinski H, Kolb D, Hermann S, Koning RI, Kohlwein SD. A role for seipin in lipid droplet dynamics and inheritance in yeast. J Cell Sci 2011; 124:3894-3904; PMID:22100922; http://dx.doi.org/10.1242/jcs.091454 22100922

[25] Szymanski KM, Binns D, Bartz R, Grishin NV, Li WP, Agarwal AK, Garg A, Anderson RG, Goodman JM. The lipodystrophy protein seipin is found at endoplasmic reticulum lipid droplet junctions and is important for droplet morphology. Proc Natl Acad Sci U S A 2007; 104:20890-5; PMID:18093937; http://dx.doi.org/10.1073/pnas.0704154104 18093937 PMC2409237

